# Solute carrier family 35 member A2 (SLC35A2) is a prognostic biomarker and correlated with immune infiltration in stomach adenocarcinoma

**DOI:** 10.1371/journal.pone.0287303

**Published:** 2023-07-19

**Authors:** Zigao Huang, Hong Yang, Jingmao Lao, Wei Deng

**Affiliations:** 1 Department of Gastrointestinal Surgery, The First People’s Hospital of Qinzhou, Qinzhou, Guangxi Zhuang Autonomous Region, China; 2 Department of Vascular Surgery, The First People’s Hospital of Qinzhou, Qinzhou, Guangxi Zhuang Autonomous Region, China; The First Affiliated Hospital of Nanjing Medical University, CHINA

## Abstract

**Background:**

Solute carrier family 35 member A2 (SLC35A2) located on the X chromosome is considered involved in the UDP-galactose transport from cytosol to Golgi apparatus and endoplasmic reticulum. It has been reported that the SLC35A2 expression is associated with carcinogenesis in recent studies, however, its specific roles in cancer progression have not been exhaustively elucidated. Herein, a system analysis was conducted to evaluate the role of SLC35A2 in prognostic, and immunology in stomach adenocarcinoma (STAD).

**Methods:**

The TIMER, GEPIA, UALCAN, Kaplan–Meier Plotter were employed to explore the SLC35A2 expression pattern and prognostic value in STAD. Genomic alterations were searched through the MEXPRESS and cBioPortal platforms. The LinkedOmics, GEPIA and Metascape databases were employed to explore the biological processes. The TIMER and TISIDB websites were utilized to investigate the relationships between SLC35A2 expression and immune cell infiltration. The associations between SLC35A2 expression and tumor mutational burden (TMB), microsatellite instability (MSI) in pan-cancer were explored using the SangerBox database.

**Results:**

Compared to the normal gastric mucosa, SLC35A2 expression was significantly increased in STAD tissues, accompanied by the robust relationships with tumor grade, histological subtypes, TP53 mutation status, TMB and prognosis. SLC35A2 and its co-expression genes played the primarily roles in purine metabolism and purinosome, including the asparagine N-linked glycosylation, protein processing in endoplasmic reticulum, regulation of transcription involved in G1/S transition of mitotic cell cycle, with the potential to participate in the regulation of VEGFA-VEGFR2 signaling pathway. Concurrently, SLC35A2 expression was correlated with macrophages and CD4+T lymphocytes infiltration in STAD.

**Conclusions:**

Our study has proposed that SLC35A2 correlated with immune cell infiltration could serve as a prognostic biomarker in STAD.

## Introduction

Stomach adenocarcinoma (STAD) lies the fifth most malignant tumor and the third most fatal cancer worldwide. Approximately 1 million STAD patients were newly diagnosed in 2018, of which over 783,000 died of this disease [1]. With the increasing development of medical and health services, more diversified methods were employed to treat cancer, including surgical resection, chemotherapy, radiotherapy, targeted therapy and hormone therapy, etc. At present, comprehensive treatment centering on chemotherapy can achieve an effectively prolonged overall survival time of patients with advanced STAD. Even so, the prognosis of STAD patients remains unoptimistic, considering the median survival time below 1 year, and the absence of effective biomarkers to guide the selection of chemotherapy for this disease [2]. As a result, it will be a challenge to identify a more effective treatments for advanced STAD in terms of current medicine. As the National Comprehensive Cancer Network guidelines state, systematic treatment is required for patients with unresectable advanced gastric cancer with the expectation of preserving major organ function and improving the life quality of patient. In recent years, emerging immunotherapies have been employed as an optimized treatment for various cancers, as well as the STAD. Previous studies have confirmed the chemotherapy combined with immunosuppressants as an effective approach to improve the prognosis of patients with advanced gastric cancer [3,4]. Unfortunately, the data on immune-related genes predicting patient survival and immunotherapy outcomes are relatively limited.

Solute carrier family 35 member A2 (SLC35A2), a protein encoding gene originally identified on Xp11.23 chromosome, mainly plays a role in the biological processes of UDP-galactose transport in the cytoplasm, such as the galactosylation of N- and O-glycans on glycoproteins in the Golgi apparatuses [5], and the synthesis of galactosylamide in the endoplasmic reticulum [6]. Studies have demonstrated a close relation of the pathogenic variation of SLC35A2 gene to a congenital disorder of glycosylation (CDG), resulting in metabolic disorders of glycan biosynthesis or assembly [7]. SLC35A2-CDG exerts an extensive impact on the functional status of many organs in patients, followed by the main clinical manifestations of neurological epilepsy symptoms, as well as the occurrence of individual growth retardation, skeletal deformity, abnormal liver function, congenital cardiac disease and other symptoms [7–9]. More recently, a study of SLC transmembrane transporter and cytotoxic drug resistance demonstrated the increased dependence of cisplatin on the transporter SLC35A2/SLC38A5 resulting from the loss of the transporter [10]. Ta Khoa and his colleagues have studied the prognostic value of SLC35 family in breast cancer (BRCA) accompanied by the correlation with tumor immune invasion [11], illustrating that SLC35A2 expression was not only associated with poor recurrence-free survival rate, but also the macrophage and neutrophil infiltration levels and multiple subtypes of BCRA, supporting the potential role of SLC35A2 in the development of various cancers. However, the SLC35A2 expression, prognostic value, immune infiltration and possible regulatory mechanisms in STAD remains largely unexplored.

To achieve a more comprehensive understanding of the relationship between SLC35A2 and the immune microenvironment in STAD, we carried out a comprehensive online analysis through multiple online databases. Our data implied SLC35A2 to contribute to the progression of STAD and the improved patient outcomes through its interaction with infiltrating immune cells.

## Materials and methods

### TIMER, TISIDB

TIMER (https://cistrome.shinyapps.io/timer/) [12,13], a comprehensive online platform, was employed for the systematical analysis, covering the expression profiles of SLC35A2 in pan-cancer, the association of the abundance of immune cell infiltration (B cells, CD4+ T cells, CD8+ T cells, Neutrophils, Macrophages, and Dendritic cells) with the expression or prognosis of SLC35A2 in STAD. Moreover, the infiltration levels among STAD with different copy number alterations of SLC35A2 was compared according to the analysis using SCNA module, mainly including deep deletion, arm-level deletion, diploid/normal, arm-level gain and high amplification. In this part, the SCNA module was focused on the difference between the infiltration level for each SCNA category and the normal level depending on the two-sided Wilcoxon rank-sum test.

To further explore the potential role of SLC35A2 in manipulating the immune invasion in STAD patients, the TISIDB database (http://cis.hku.hk/TISIDB/) integrating multiple heterogeneous data types for tumor and immune system interactions was utilized to analyze the correlation between the abundance of 28 tumor-infiltrating lymphocytes and the expression level of SLC35A2 [14]. At the same time, TISIDB provided the distribution of SLC35A2 expression across immune and molecular subtypes.

### GEPIA, UALCAN, SangerBox

The GEPIA and UALCAN online analysis platform were employed to achieve a comprehensive analysis to examine SLC35A2 gene expression difference between STAD tissue and normal gastric mucosa. Gene Expression Profiling Interactive Analysis (GEPIA)(http://gepia.cancer-pku.cn/) [15] was used to determine the expression pattern of given genes in STAD tissues and normal gastric mucosa, which serves as an interactive web tool for analyzing the target gene expression profiles extracted from the TCGA and the GTEx database. The |Log2FC| Cutoff and p-value Cutoff were set as 1, 0.01, respectively. Additionally, GEPIA also allows options for examining the association of the expression of given genes with STAD patient survival, covering the overall survival (OS) and disease-free survival (DFS). The HR and P values of log-rank test results were directly plotted.

For the further verification of the expression pattern of SLC35A2 on GEPIA website, UALCAN (http://ualcan.path.uab.edu/index.html) [16] was applied to exploring the differences in expression level of SLCA35A2 target gene between STAD tissue and normal gastric mucosa, which serves as an interactive web resource for analyzing cancer OMICS data, allowing the expression of target genes across multiple dimensions in multiple cancers, covering RNA expression, methylation, and protein levels. In the present study, this user-friendly online tool was also employed to identify the effects of different clinicopathological features on SLC35A2 gene expression level in STAD tissues, such as race, gender, tumor grade, histological subtypes, TP53 mutation status. SangerBox (http://sangerbox.com), an online tool for TCGA analysis, was employed to investigate the association between SLC35A2 expression and tumor mutational burden (TMB), microsatellite instability (MSI) in pan-cancer.

### Kaplan–Meier plotter

Kaplan–Meier plotter (https://kmplot.com/analysis/) [17] was applied to analyze the potential prognostic value of SLC35A2 in patients with STAD, which served as an open-source project to explore the correlation between 30,000 gene expressions and survival in 21 tumor types, including gastric cancer. According to the best separation of SLC35A2 expression, patients were divided into “high” and “low” risk groups, the log-rank test was employed for comparison of survival differences among the two groups, with the log-rank P-value and hazard ratio (HR) recorded. Concurrently, the Kaplan–Meier plotter was utilized to investigate the correlations between SLC35A2 expression and OS, first-progression (FP), and post-progression survival (PPS) in STAD patients according to the different clinical characteristics, covering stage, lauren classification, differentiation, gender, perforation and so on.

### MEXPRESS, cBioPortal

To evaluate the SLC35A2 DNA methylation and alterations in STAD, a combined analysis was performed applying multiple online databases. Based on the samples from TCGA, MEXPRESS (https://mexpress.be/index.html) [18] as an intuitive online web tool for the visualization and interpretation offered the promoter methylation status of the SLC35A2. Another prominent feature of this tool is that it allows an easy availability of the information and relationship between the expression of the target gene, DNA methylation and clinical data. In this study, the target gene "SLC35A2" was directly input to select the tumor type "STAD" on the left side of the website. Afterwards, the system conducted person test based on 544 STAD samples and displayed the statistical results. Furthermore, the genetic alteration analysis was applied on cBioPortal website (http://www.cbioportal.org/) [19], an online data mining platform, which allows the quantification of the mutation rates and distributions of SLC35A2 or its related genes based on TCGA data.

### LinkedOmics, Metascape

LinkedOmics (http://www.linkedomics.org/admin.php) [20], a data analysis platform covering over 32 cancer types of multi-omics, was applied to identifying 100 co-expressed genes of SLC35A2 via Pearson Correlation test in STAD dataset, which were presented in heat maps and volcano maps. Subsequently, the top 100 genes most similar of SLC35A2 were screened out from GEPIA database, and the expression, prognosis and mutation frequency of the co-expressed and most similar genes in STAD were examined through multiple databases, such as UALCAN, cBioPortal and GEPIA. In addition, these 200 related-expressed genes were loaded into the Metascape website (https://metascape.org) [21] for Gene Ontology (GO) and Kyoto Encyclopedia of Genes and Genomes (KEGG) pathway analysis.

## Results

### Expression levels of SLC35A2 in STAD

**Fig 1:**
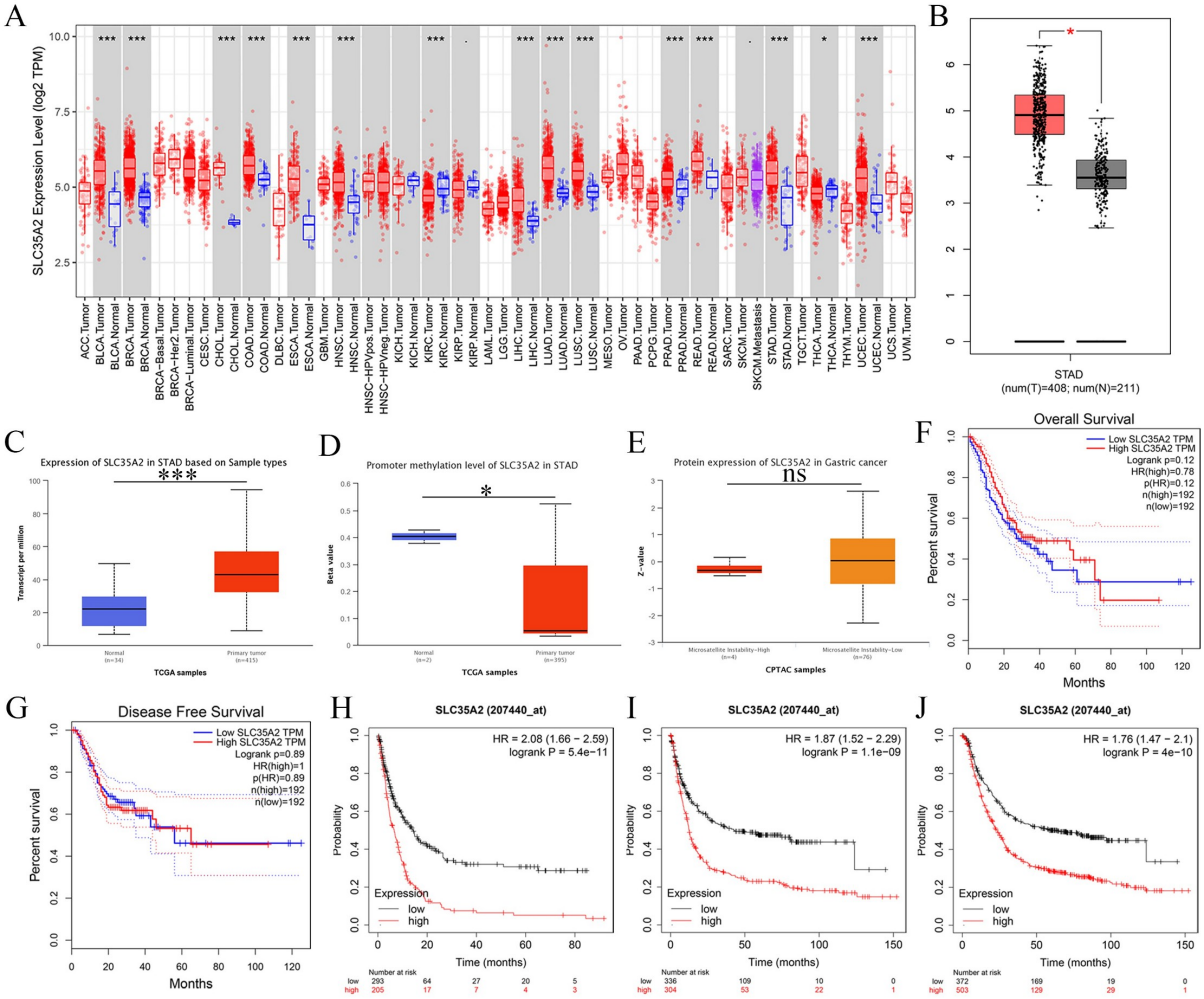
Expression pattern and prognostic value of SLC35A2 in STAD. (A) Differences in the expression of SLAC35A2 in cancer tissues and normal tissues (TIMER 2.0). (B) Differences in the expression of SLAC35A2 in STAD cancer tissues and normal tissues (GEPIA). (C–E) Differences in the expression, promoter methylation, and protein levels of SLC35A2 in STAD primary tumor and normal tissues, respectively (UCLCAN). (F, G) Difference of SLC35A2 expression in OS, DFS of STAD patients (GEPIA). (H–J) Difference of SLC35A2 expression in OS, FP, PPS of STAD patients, respectively (Kaplan–Meier Plotter).

To evaluate the overall expression pattern of SLC35A2 in pan-cancer, we first employed the TIMER database to analyze the difference between tumors tissues and normal tissues, revealing the significantly up-regulated SLC35A2 gene expression in most tumor types compared with normal tissues, including the BLCA, BRCA, CHOL, COAD, ESCA, HNSC, LIHC, LUAD, LUSC, PRAD, READ, UCEC. The down-regulation of SLC35A2 was found in only two tumor tissues (KIRC, THCA), while no change was revealed in the expression of other tumors (KICH, KIRP and so on) (Fig 1A). Notably, the expression of SLC35A2 gene was higher in STAD tissues compared to the normal tissues (P<0.05), which was also further verified by the analysis of GEPIA (Fig 1B). We further assessed the differences in SLC35A2 expression using the UALCAN database, as well as the promoter methylation, and protein levels between STAD primary tumor and normal tissues. Our results suggested the over-expressed SLC35A2 at mRNA level while the down-expression at methylation level in primary tumors compared with normal tissues. No statistical significance was revealed on the protein expression of SLC35A2 in STAD between primary tumor tissue and normal tissue (Fig 1C–1E). The findings above suggested a potential role of SLC35A2 in the pathogenesis and development of STAD, which deserves further study.

### Prognostic value of SLC35A2 in STAD

The prognostic value of SLC35A2 expression was analyzed depending on the GEPIA and Kaplan–Meier Plotter platforms. No statistically significant differences were found in terms of the difference between the SLC35A2 expression and patients’ OS (log-rank P = 0.12) and PFS (log-rank P = 0.89) in GEPIA (Fig 1F and 1G), nevertheless, up-regulation of SLC35A2 predicted worse OS (HR = 2.08(1.66–2.59), log-rank P = 5.4e-11), FP (HR = 1.87(1.52–2.29), log-rank P = 1.1e-09), and PPS (HR = 1.76(1.47–2.1), log-rank P = 4e-10) in STAD patients based on Kaplan–Meier Plotter (Fig 1H–1J). Meanwhile, among the stage 2 patients, the SLC35A2 expression was found significantly correlated to clinical outcomes, that were, OS (HR = 2.44 (1.34–4.45), P = 0.0027), FP (HR = 2 (1.1–3.66), P = 0.021), and PPS (HR = 2.6 (1.35–5.04), P = 0.0032). And the Kaplan-Meier Plotter was utilized to analyze the association of SLC35A2 expression level and prognostic employing different clinical characteristics, such as stage, stage T, stage N, stage M, and lauren classification, and with the results detailed in Table 1. To sum up, these results obviously demonstrated a significantly relation of the higher SLC35A2 expression with the worse outcomes in STAD patients, and that SLC35A2 might serve as an independent prognostic biomarker in STAD patients.

**Table 1:** Correlation between SLC35A2 expression and prognostic based on different clinical characteristics using Kaplan–Meier Plotter.

Factors	n	OS		FP		PPS	
		Hazard ratio	P-value	Hazard ratio	P-value	Hazard ratio	P-value
Stage							
1	69	3.24(1.03–10.21)	0.034	2.29(0.69–7.63)	0.17	2.24(0.5–10.13)	0.28
2	145	2.44(1.34–4.45)	0.0027	2(1.1–3.66)	0.021	2.6(1.35–5.04)	0.0032
3	319	1.82(1.32–2.51)	0.00024	1.71(1.17–2.49)	0.0047	2.07(1.34–3.21)	0.00081
4	152	1.77(1.19–2.63)	0.0041	1.69(1.1–2.59)	0.015	2.12(1.31–3.42)	0.0018
Stage T							
1	14	-	-	-	-	-	-
2	253	1.73(1.12–2.68)	0.012	1.57(1.03–2.4)	0.035	2.07(1.31–3.29)	0.0015
3	208	1.86(1.29–2.67)	0.00066	1.67(1.18–2.37)	0.0038	1.85(1.25–2.74)	0.0018
4	39	2.02(0.8–5.12)	0.13	2.55(1.12–5.8)	0.02	1.81(0.51–6.37)	0.35
Stage N							
0	78	2.98(1.28–6.69)	0.0082	2.99(1.29–6.97)	0.0077	3.24(1.02–10.28)	0.035
1+2+3	437	1.99(1.53–2.59)	1.70E-07	1.82(1.41–2.34)	2.70E-06	2.2(1.65–2.93)	2.90E-08
1	232	2.48(1.63–3.76)	1.00E-05	2.2(1.48–3.27)	6.60E-05	3.31(2.1–5.22)	5.70E-08
2	129	1.79(1.13–2.86)	0.013	1.69(1.09–2.6)	0.017	1.82(1.1–3)	0.018
3	76	2.36(1.36–4.1)	0.0016	2.12(1.23–3.65)	0.0059	2.02(1.12–3.62)	0.017
Stage M							
0	459	1.95(1.48–2.58)	1.70E-06	1.77(1.36–2.31)	2.10E-05	2.39(1.77–3.22)	4.50E-09
1	58	3.28(1.68–6.43)	0.00029	2.03(1.04–3.96)	0.034	4.63(1.8–11.93)	0.00057
Lauren classification							
instestinal	336	2.41(1.74–3.33)	4.40E-08	2.02(1.4–2.93)	0.0014	3.18(2.09–4.82)	1.20E-08
diffuse	248	1.71(1.21–2.41)	0.002	1.7(1.2–2.39)	0.0023	1.6(1.1–2.34)	0.014
mixed	33	2.45(0.84–7.11)	0.09	1.76(0.59–5.26)	0.31	-	-
Differentiation							
poor	166	1.21(0.79–1.84)	0.38	1.53(0.9–2.6)	0.12	3.13(1.5–6.57)	0.0015
moderate	67	2.63(1.07–6.49)	0.03	2.97(1.22–7.26)	0.013	0.24(0.08–0.75)	0.0083
well	32	3.77(1.1–12.87)	0.023	-	-	-	-
Gender							
female	244	2.25(1.53–3.29)	2.00E-05	2.19(1.48–3.24)	6.30E-05	2.33(1.51–3.59)	8.30E-05
male	566	1.82(1.45–2.27)	1.20E-07	1.81(1.42–2.3)	1.20E-06	2.03(1.56–2.63)	6.50E-08
Perforation							
no	169	1.49(0.97–2.28)	0.069	1.4(0.93–2.13)	0.11	2(1.14–3.52)	0.014
yes	4	-	-	-	-	-	-
Treatment							
surgery alone	393	1.51(1.13–2.02)	0.0047	1.39(1.05–1.84)	0.02	1.89(1.38–2.59)	4.90E-05
5 FU based adjuvant	157	0.75(0.52–1.08)	0.13	0.83(0.57–1.22)	0.34	0.52(0.35–0.76)	0.00066
other adjuvant	80	2.25(0.93–5.44)	0.064	2.24(1.01–4.94)	0.041	2.19(0.89–5.37)	0.08
HER2 status							
HER2 negative	641	1.88(1.49–2.37)	5.80E-08	1.91(1.47–2.47)	6.80E-07	2.04(1.54–2.72)	5.60E-07
HER2 positive	424	1.48(1.1–1.98)	0.0082	1.7(1.2–2.42)	0.0024	2(1.33–2.99)	0.00066

### Relationship between SLC35A2 expression and clinical factors

**Fig 2:**
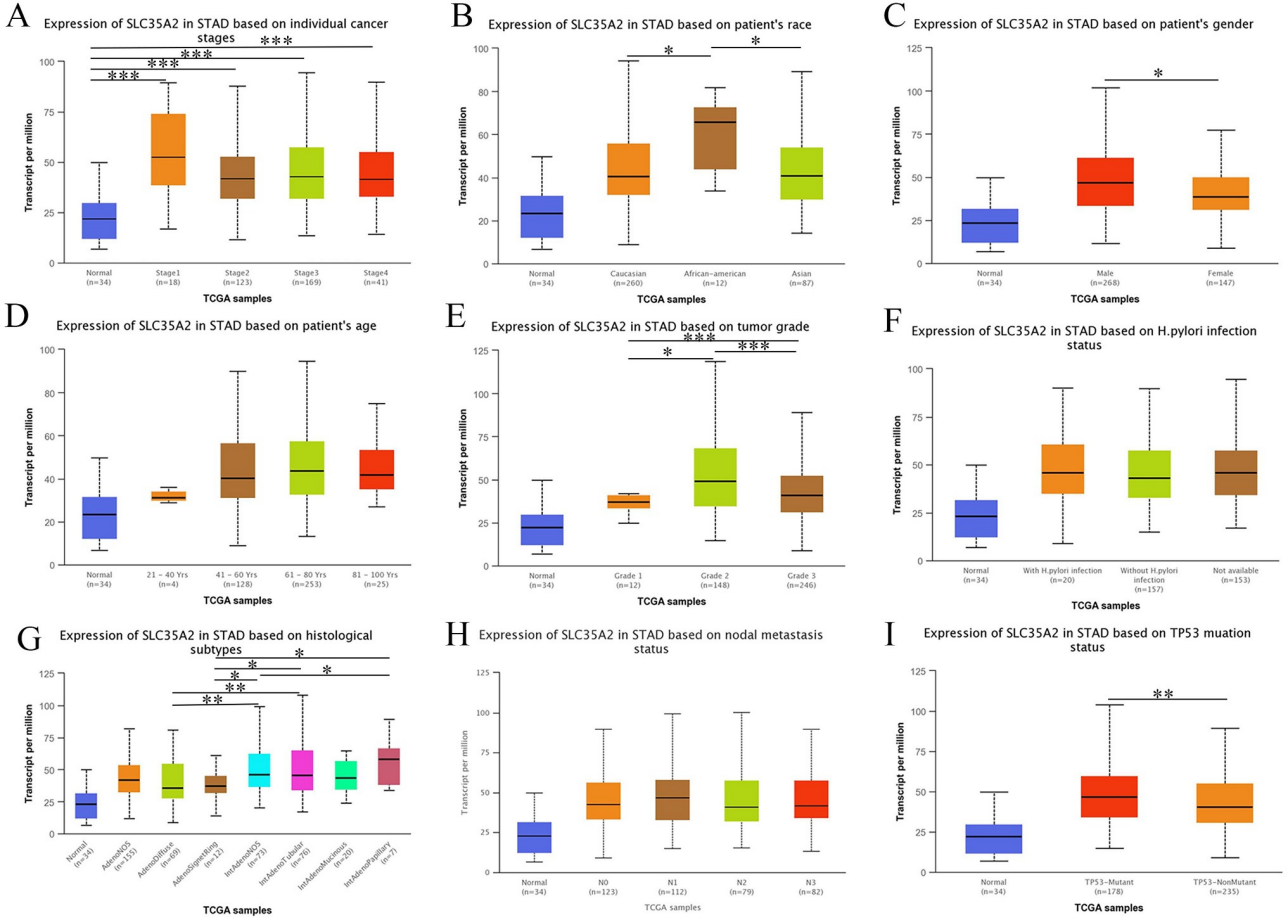
Effects of clinicopathological factors on SLAC35A2 expression. (A–I) Relevance of SLC35A2 expression level and individual cancer, race, gender, age, tumor grade, H.pylori infection, histological subtypes, metastasis status and TP53 mutation status.

To achieve a further illustration on the role of SLC35A2 in the progression of STAD, we employed multiple online databases to explore the correlation between SLC35A2 expression level and clinicopathological features using. At first, the results on GEPIA and TISIDB websites indicated the influence of different immune subtypes (C1, C2, C3, C4, C6, P = 1.17e-08) and molecular subtypes (CIN, EBV, GS, HM-SNV, HM-indel, P = 1.6e-08) the independence of on SLC35A2 expression, despite its independence of tumor stage (P = 0.196) (S1 Fig). Additionally, we performed a subgroup analysis to explore the correlation of SLC35A2 expression level with multiple clinicopathological features, such as gender, histological subtypes, TP53 mutation status (Fig 2A–2I).

In most human cancers, such as STAD, STES, BLCA, PCPG, LUAD, MESO, SLC35A2 expression was found to exhibit significant positive associations with TMB, and negative correlations in COAD, LAML. MSI analysis revealed the positive associations of SLC35A2 expression with KIPAN, SARC, GBM, CHOL, and the negative with COAD, GBMLCG, DLBC. The abnormal expression of SLC35A2 displayed no correlation with the increased mutation of MMR gene in STAD (S2 Fig).

### Promoter methylation and genetic alterations levels of SLC35A2 in STAD

**Fig 3:**
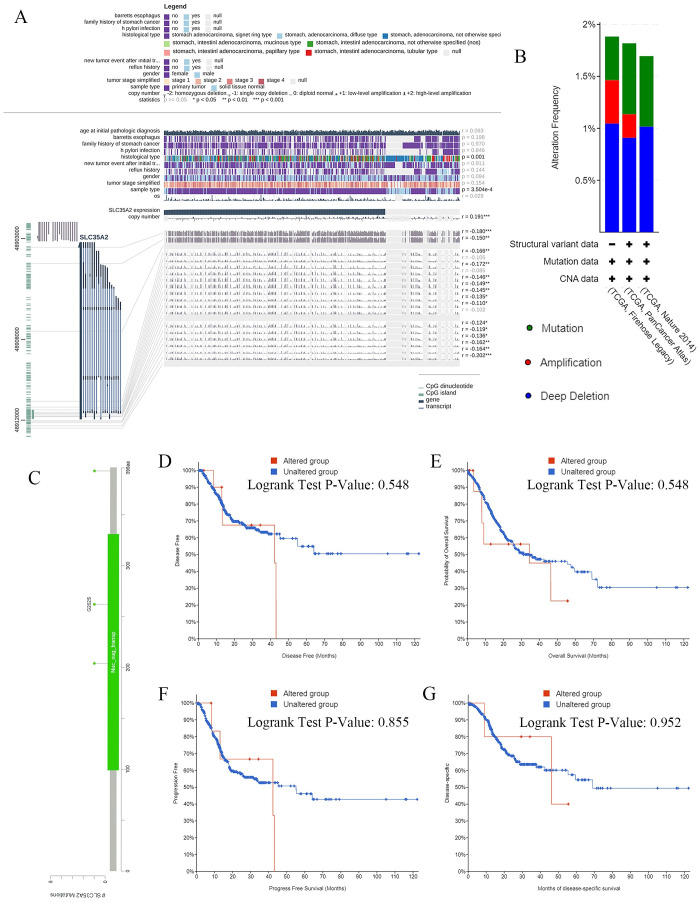
Promoter methylation and genetic alterations levels of SLC35A2 in STAD. (A) Relationships between SLC35A2 expression and copy number, clinical data (MEXPRESS). (B) Alteration frequency of SLC35A2 in different STAD studies. (C) Mutation diagram providing information on the mutation sites, mutation types, and the number of cases, the results were colored with respect to the corresponding mutation types. (D) Associations between SLC35A2 mutations and patient outcomes, including DFS, OS, PFS, and DSS.

After determining the potential prognostic value of SLC35A2 in STAD patients, we further explored the promoter methylation and genetic alterations levels of SLC35A2, to elucidate its relationship with the occurrence and development of cancer. To begin with, MEXPRESS was employed to obtain the data about the relationships of SLC35A2 expression with copy number, clinical data (age at initial pathologic diagnosis, barrettes esophagus, family history of stomach cancer and so on), which revealed a significant relation of SLC35A2 expression to histological type (P = 0.001), sample type (P = 3.504e-4), as well as a strong correlation with the copy number (r = 0.191, P < 0.05) (Fig 3A).

Next, the SLC35A2 genetic alterations in three STAD cohorts were further explored using the cBioPortal tool. In brief, the analysis on 1213 STAD patients from TCGA suggested the higher frequency of deep deletion compared to mutation and amplification among TCGA Nature 2014, TCGA PanCancer Atlas, and TCGA Firehose Legacy (Fig 3B). The genomic alteration types and the location of distinct mutations were detailed in Fig 3C, which indicated a total of 7 mutation sites identified in SLC35A2 that were located between amino acids 0 and 396. While no associations between SLC35A2 mutations and patient outcomes were found, including DFS (P = 0.548), OS (P = 0.548), PFS (P = 0.855), and DSS (P = 0.952) (Fig 3D–3G).

### Functional enrichment analyses of SLC35A2

**Fig 4:**
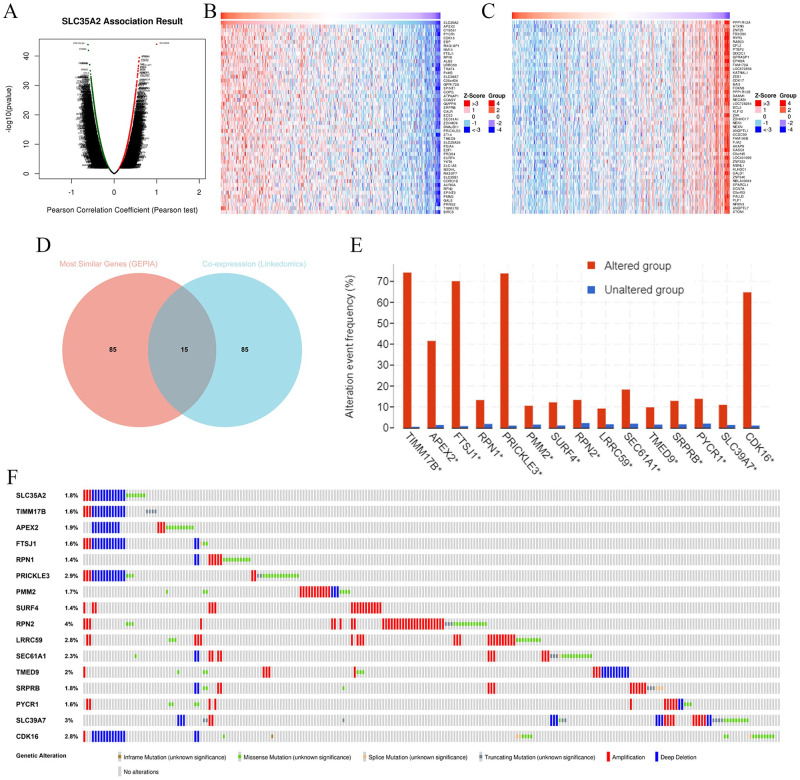
Co-expression networks of SLC35A2 gene. (A) The positive and negative related genes showed by volcano map. (B, C) The top 50 positive and negative related genes of SLC35A2 gene listed in heat maps, respectively. (D) Co-expressed genes between 100 most similar genes of SLC35A2 from GEPIA and the 100 co-expressed network genes from LinkedOmics. (E) Expression pattern of 15 co-expressed genes between altered group and unaltered group. (F) Mutation frequency of 15 co-expressed genes.

**Fig 5:**
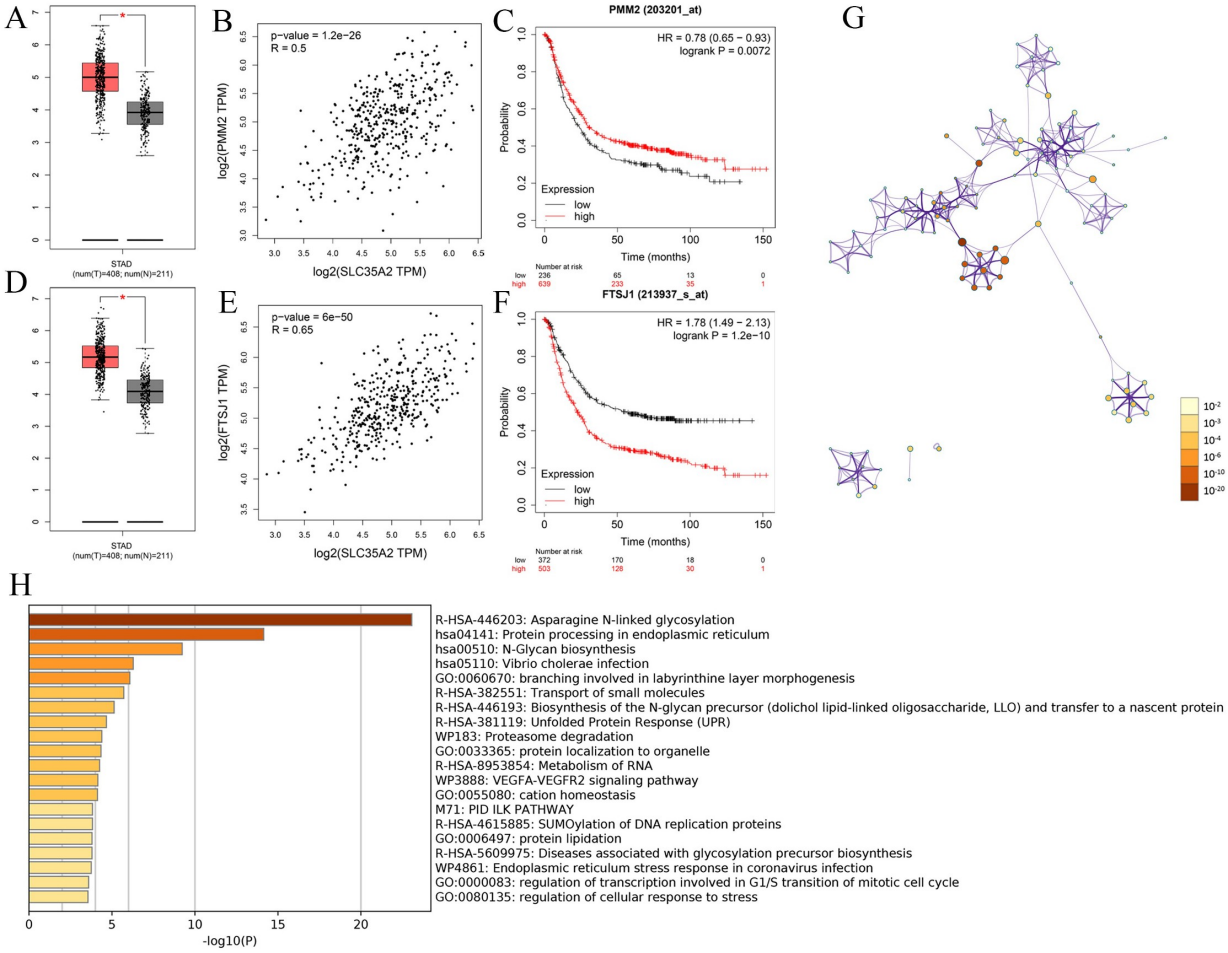
Functional enrichment analyses of SLC35A2. (A) Expression pattern of PMM2 in STAD cancer and normal tissue. (B) Relationship between PMM2 and SLC35A2 in STAD. (C) Prognostic value of PMM2 in STAD. (D) Expression pattern of FTSJ1 in STAD cancer and normal tissue. (E) Relationship between FTSJ1 and SLC35A2 in STAD. (F) Prognostic value of FTSJ1 in STAD. (G) Nodes in the same enrichment network colored by P value, as shown in the legend, where the darker color represented the more significant node (see legend for P value ranges). (H) The enrichment of SLC35A2–related genes mainly in asparagine N-linked glycosylation, protein processing in endoplasmic reticulum, metabolism of RNA, etc. showed by Go analysis.

To understand the potential biological functions of SLC35A2 in STAD, several online analytical portals were employed for investigation. To start with, the co-expression networks of SLC35A2 gene were constructed via LinkedOmics. The volcano map indicated the related genes of SLC35A2 association, positive or the negative correlation (Fig 4A), which revealed the top 50 positive and negative related genes of SLC35A2 gene, respectively (Fig 4B and 4C). PPP1R12A, ATXN3, APEX2 showed the strongest associations with SLC35A2 expression (Person correlation = -6.155e-01, -6.042e-01, 5.895e-01 and P-value = 1.280e-44, 1.193e-42, 3.378e-40). Among the 100 most related genes of SLC35A2 from GEPIA and the 100 co-expressed network genes from LinkedOmics, 15 co-expressed genes (TIMM17B, APEX2, FTSJ1, RPN1, PRICKLE3, PMM2, SURF4, RPN2, LRRC59, SEC61A1, TMED9, SRPRB, PYCR1, SLC39A7, CDK16) were identified (Fig 4D), which exhibited obvious differences in expression between cancer and normal tissues (Fig 4E). Of these genes, RPN2 and SLC39A7 displayed the mutation frequencies of 4% and 3%, respectively (Fig 4F). Afterwards, the GEPIA and Kaplan–Meier Plotter databases were employed to evaluate the relationship of two co-expressed genes (FTSJ1, PMM2) with the expression of the target gene SLC35A2 in STAD, which showed the strong correlation of FTSJ1 and PMM2 genes with SLC35A2 with high expression in STAD tissues. The high expression of PMM2 was closely associated with the better OS in patients; conversely, the upregulation of FTSJ1 expression may predict the opposite (Fig 5A–5F), which may be attributed to the different sources of analyzed samples.

To further investigate the functions and pathways enriched in SLC35A2-related genes, SLC35A2-related genes from GEPIA and LinkedOmics databases were enrolled into Metascape analysis platform for GO and KEGG pathway analysis (Fig 5G and 5H). The results demonstrated the enrichment of SLC35A2 co-expression genes mainly in asparagine N-linked glycosylation, protein processing in endoplasmic reticulum, metabolism of RNA, VEGFA-VEGFR2 signaling pathway, SUMOylation of DNA replication proteins, diseases associated with glycosylation precursor biosynthesis, endoplasmic reticulum stress response in coronavirus infection, regulation of transcription involved in G1/S transition of mitotic cell cycle, regulation of cellular response to stress.

### Correlation between SLC35A2 expression and immune infiltrating cells

**Fig 6:**
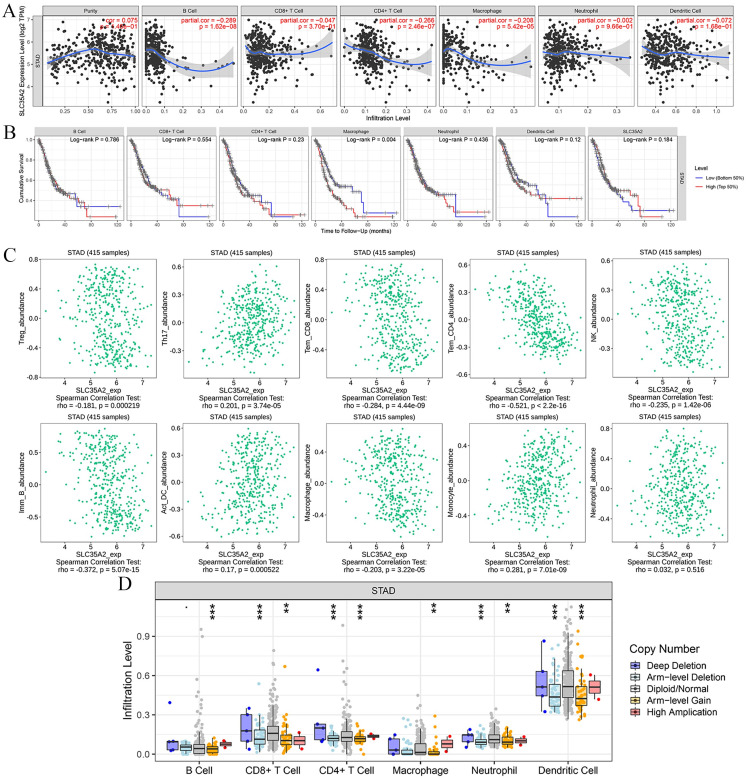
Correlation between SLC35A2 expression and immune infiltrating cells. (A) Relationship between the SLC35A2 expression level and the immune cell infiltration level (TIMER 2.0). (B) Relationship between immune cell abundance and survival of patients with STAD. (C) Relationship between the SLC35A2 expression level and the immune cell infiltration level (TISIDB). (D) Link between immune infiltrates and somatic CNV, including the deep deletion, arm-level deletion, diploid/normal, arm-level gain, high amplication.

By employing the "Correlation" module of TIMER analysis platform, we explored the correlation of SLC35A2 gene expression level with tumor infiltrating immune cell density and cell-specific markers. On the one hand, significant correlation was revealed of the expression of SLC35A2 with the infiltration level of B cells (partial.cor = -0.289, p = 1.62e-08), CD4+ T cells (partial.cor = -0.266, p = 2.46e-07), macrophages (partial.cor = -0.208, p = 5.42e-05), but not with purity tumor (partial.cor = 0.075, p = 1.46e-01), CD8+ T cells (partial.cor = -0.047, p = 3.7e-01), neutrophils (partial.cor = -0.002, p = 9.66e-01) and dendritic cells (partial.cor = -0.047, p = 3.7e-01), unfortunately (Fig 6A). Immune cells as the critical components of immune microenvironment can significantly affect the survival of cancer patients. We further examined whether the high levels of immune cell infiltration were associated with longer survival in STAD patients. And no significant correlation with survival time was found in B cells (log-rank P = 0.786), CD8+ T cells (log-rank P = 0.554), CD4+ T cells (log-rank P = 0.23), neutrophils (log-rank P = 0.004) P = 0.436), dendritic cells (log-rank P = 0.12) and SLC35A2 (log-rank P = 0.184), in addition to macrophages (log-rank P = 0.004) (Fig 6B). The correlations between SLC35A2 and gene markers of various immune cells via TIMER were provided in Table 2.

**Table 2:** Correlation between SLC35A2 and gene markers of various immune cells via TIMER.

Description	Gene markers	None		Tumor Purity	
		cor	p	partial.cor	p
B cell	CD19	-0.172	***4*.*33E-04***	-0.149	***3*.*65E-03***
	CD79A	-0.162	***9*.*45E-04***	-0.138	***7*.*34E-03***
CD8+ T cell	CD8A	-0.085	8.84E-02	-0.052	3.14E-01
	CD8B	-0.066	1.76E-01	-0.024	6.47E-01
Dendritic cell	ITGAX	0.11	***2*.*53E-02***	0.134	***9*.*25E-03***
	NRP1	0.036	4.65E-01	0.025	6.27E-01
	CD1C	-0.23	***2*.*10E-06***	-0.245	***1*.*37E-06***
	HLA-DPA1	0.016	7.45E-01	0.053	3.05E-01
	HLA-DRA	0.012	8.02E-01	0.05	3.31E-01
	HLA-DQB1	0.031	5.29E-01	0.069	1.79E-01
	HLA-DPB1	-0.035	4.73E-01	-0.003	9.57E-01
M1 Macrophage	PTGS2	0.004	9.31E-01	0.007	8.89E-01
	IRF5	0.173	***3*.*87E-04***	0.188	***2*.*37E-04***
	NOS2	0.022	6.51E-01	0.044	3.95E-01
M2 Macrophage	MS4A4A	-0.029	5.50E-01	-0.026	6.12E-01
	VSIG4	0.024	6.20E-01	0.027	5.96E-01
	CD163	0.091	6.26E-02	0.092	7.25E-02
Monocyte	CSF1R	0.055	2.67E-01	0.063	2.20E-01
	CD86	0.017	7.34E-01	0.053	3.06E-01
Natural killer cell	KIR2DS4	-0.045	3.63E-01	-0.018	7.31E-01
	KIR3DL3	0.089	7.11E-02	0.131	***1*.*06E-02***
	KIR3DL2	-0.091	6.36E-02	-0.051	3.21E-01
	KIR3DL1	-0.077	1.16E-01	-0.054	2.93E-01
	KIR2DL4	0.04	4.20E-01	0.072	1.60E-01
	KIR2DL3	-0.115	***1*.*87E-02***	-0.088	8.69E-02
	KIR2DL1	-0.079	1.08E-01	-0.037	4.70E-01
Neutrophils	CCR7	-0.154	***1*.*66E-03***	-0.127	***1*.*34E-02***
	ITGAM	0.076	1.23E-01	0.077	1.37E-01
	CEACAM8	-0.029	5.61E-01	-0.012	8.17E-01
T cell (general)	CD3D	-0.144	***3*.*25E-03***	-0.103	***4*.*48E-02***
	CD3E	-0.112	***2*.*28E-02***	-0.067	1.96E-01
	CD2	-0.119	***1*.*49E-02***	-0.078	1.28E-01
T cell exhaustion	CTLA4	0.043	3.81E-01	0.098	5.61E-02
	LAG3	0.046	8.53E-01	0.102	***4*.*66E-02***
	HAVCR2	0.074	1.33E-01	0.096	6.11E-02
	GZMB	0.083	9.10E-02	0.146	***4*.*42E-03***
	PDCD1	0.046	3.46E-01	0.103	***4*.*47E-02***
TAM	CCL2	-0.101	***3*.*88E-02***	-0.093	7.10E-02
	IL10	0.047	3.41E-01	0.071	1.65E-01
	CD68	0.284	***3*.*72E-09***	0.304	***1*.*58E-09***
Tfh	BCL6	0.284	***3*.*72E-02***	0.304	***1*.*58E-09***
	IL21	0.025	6.17E-01	0.057	2.64E-01
Th1	TBX21	-0.047	3.41E-01	0.004	9.39E-01
	STAT4	-0.15	***2*.*11E-03***	-0.113	***2*.*75E-02***
	STAT1	0.205	***2*.*56E-05***	0.248	***9*.*71E-07***
	IFNG	0.046	3.46E-01	0.107	***3*.*67E-02***
	IL13	0.063	1.99E-01	0.093	7.15E-02
Th2	GATA3	-0.152	***1*.*95E-03***	-0.114	***2*.*70E-02***
	STAT6	0.171	***4*.*78E-04***	0.158	***2*.*06E-03***
	STAT5A	0.093	5.95E-02	0.097	5.98E-02
Th17	STAT3	0.221	***2*.*69E-06***	0.209	***3*.*95E-05***
	IL17A	0.183	***1*.*84E-04***	0.214	***2*.*56E-05***
Treg	FOXP3	0.107	***2*.*89E-02***	0.162	***1*.*55E-03***
	CCR8	0.085	8.42E-02	0.122	***1*.*76E-02***
	STAT5B	0.009	8.62E-01	-0.009	8.68E-01
	TGFB1	-0.031	5.27E-01	-0.013	8.03E-01

To further illustrate the potential role of SLC35A2 gene in immune cell regulation, the association of SLC35A2 expression level with lymphocyte abundance was subsequently investigated via TISIDB platform. The outcomes indicated the negative correlation of the high expression of SLC35A2 gene with the increased density infiltration of Treg cells (rho = -0.181, P = 0.000219), Th17 cells (rho = -0.201, P = 3.74e-05), Tem_CD8 cells (rho = -0.284, P = 4.44e-09), Tem_CD4 cells (rho = -0.521, P<2.2e-16), NK cells (rho = -0.235, P = 1.42e-06), Imm_B cells (rho = -0.372, P = 5.07e-15), Macrophage cells (rho = -0.203, P = 3.22e-05), and the positive correlation with the increased density infiltration of Act_DC cells (rho = 0.17, P = 0.000522), Monocyte cells (rho = 0.281, P = 7.01e-09)(Fig 6C). These relationships were detailed in Table 3.

**Table 3:** Relationship between SLC35A2 levels and 28 tumor immunoinfiltrating cell subtypes using TISIDB.

Description	rho	p
Activated CD8 T cell (Act_CD8)	-0.026	0.593
Central memory CD8 T cell (Tcm_CD8)	0.09	***0*.*0664***
Effector memeory CD8 T cell (Tem_CD8)	-0.284	***4*.*44E-09***
Activated CD4 T cell (Act_CD4)	0.052	0.289
Central memory CD4 T cell (Tcm_CD4)	-0.022	0.652
Effector memeory CD4 T cell (Tem_CD4)	-0.521	***<2*.*2e-16***
T follicular helper cell (Tfh)	-0.204	***3*.*03E-05***
Gamma delta T cell (Tgd)	-0.065	0.188
Type 1 helper cell (Th1)	-0.252	***2*.*06E-07***
Type 17 helper cell (Th17)	0.201	***3*.*74E-05***
Type 2 helper cell (Th2)	-0.247	***3*.*63E-07***
Regulatrory T cell (Treg)	-0.181	***0*.*000219***
Activated B cell (Act_B)	-0.367	***1*.*41E-14***
Immature B cell (Imm_B)	-0.372	***5*.*07E-15***
Memory B cell (Mem_B)	-0.279	***8*.*79E-09***
Natural killer cell (NK)	-0.235	***1*.*42E-06***
CD56bright natural killer cell (CD56bright)	0.205	***2*.*70E-05***
CD56dim natural killer cell (CD56dim)	0.345	***7*.*13E-13***
Myeloid derived suppressor cell (MDSC)	-0.133	***0*.*00673***
Natural killer T cell (NKT)	-0.231	***2*.*19E-06***
Activated dendritic cell (Act_DC)	0.17	***0*.*000522***
plasmacytoid dendritic cell (pDC)	0.047	0.339
Immature dendritic cell (iDC)	0.025	0.61
Macrophage (Macrophage)	-0.203	***3*.*22E-05***
Eosinophil (Eosinophil)	-0.297	***8*.*08E-10***
Mast cell (Mast)	-0.304	***3*.*01E-10***
Monocyte (Monocyte)	0.281	***7*.*01E-09***
Neutrophil (Neutrophil)	0.032	5.16E-01

Additionally, we further explored the link between immune infiltrates and somatic CNV by employing the TIMER 2.0 to, covering the deep deletion, arm-level deletion, diploid/normal, arm-level gain, high amplication, with the results presented in Fig 6D. Nevertheless, these findings may suggest the certain influences of SLC35A2 on the infiltration of immune cells in the immune microenvironment through some potential pathway in gastric cancer, including CD4+ T cell activation and macrophage polarization, which requires further work to confirm.

## Discussion

In recent years, great progress has been made on the multidisciplinary diagnosis and treatment model in tumor treatment, followed by the improved clinical treatment effect of STAD patients to a certain extent. However, due to the lack of reliable and effective early diagnostic markers, most STAD patients are faced with the advanced stage when first diagnosed, who could enjoy the five-year survival rate of only 30% [22]. As a result, these models have not fundamentally solved the diagnosis and treatment of advanced tumors like STAD, lacking the substantive answer to a series of basic questions that promote the occurrence and development of tumors, including the causes of tumorigenesis, factors of tumor metastasis, transformation of tumor microenvironment and immune depletion, have not been [23]. As we all know, a nonnegligible core direction of global cancer research lies the mechanism of tumor-related driver genes that manipulate the cancer development. Scientists have attempted to explore new tumor targets to inhibit tumor proliferation, migration and invasion, so as to modify the tumor microenvironment and activate anti-tumor immune system. Based on this assumption, we carried out a systematical investigation on the role of SLC35A2 in STAD depending on the online expression databases and bioinformatics data mining tools.

To start with, we investigated the expression profile of SLC35A2 gene in pan-cancer and its association with clinical outcomes via TIMER, GEPIA, UALCAN and Kaplan–Meier Plotter, which consistently indicated the altered SLC35A2 expression in various cancer tissues, that were BLCA, BRCA, CHOL, COAD, ESCA, HNSC, LIHC, LUAD, LUSC, PRAD, READ, UCEC. The inconsistent expression of SLC35A2 in pan-cancer may result from the diverse in the methods of data collection in different studies, or the diverse in the underlying biological mechanisms. A recent study has revealed a strong association of high expression of SLC35A2 with the decreased relapse-free survival in BRCA patients, suggesting that it to serve as a reliable prognostic marker for the treatment of BRCA [11]. In another study, Chien-Liang Liu [24] has demonstrated the gradually increased SLC35A2 expression in the cytoplasm from carcinoma in situ to invasive carcinoma, utilizing the immunohistochemical experiments, and this expression pattern also showed the correlation with the stage of BRCA. In the present report, we robustly demonstrated the significantly up-regulated expression of SLC35A2 in STAD tissues compared with normal tissues, meanwhile, it was related to a worse prognosis in STAD patients. As a result, these findings collectively elucidated the different functions SLC35A2 in oncogenesis and tumor progression in various cancers, with the potential to serve as a predictive biomarker for prognosis of STAD, which deserves further investigation. In a previous study, it has been revealed that SLC5A7 as a member of the solute carrier superfamily could promote the p53 protein expression by direct interaction with and modification on p53 and disruption on the interaction between p53 and MDM2 in wild type p53 CRC cells [25]. Interestingly, our results revealed the highly up-regulated SLC35A2 expression in STAD tissues with TP53 mutation compared to those without TP53 mutation, which suggests that SLC35A2 may be involved in the carcinogenesis process of the TP53 pathway.

Increasing studies have confirmed the inextricable relation of TMB and MSI to the occurrence and development of cancer, which are commonly adopted in clinical evaluation of the therapeutic effect of immune checkpoint inhibitors [26–28]. In the present work, the correlations between SLC35A2 expression and TMB, MSI were investigated. As indicated by our result, SLC35A2 expression exhibited significantly positive associations with TMB in STAD, revealing the higher potential of patients to benefit from tumor immunotherapy in line with the higher expression of SLC35A2 in STAD. In terms of MSI, tumors with a high mutation load may encode non-autoimmunogenic new epitopes, activating lymphocyte recruitment within the tumor and inducing a strong immune response [29]. Unfortunately, our results failed to suggest the relation of the aberrant expression of SLC35A2 to the promoted mutation of MMR gene in STAD. Anyway, the research results above require further verification.

Solute carrier transporters serve as the largest family of transporters in human body, covering liver, kidney and other organs [30,31]. They exert the physiological functions by transporting glucose, amino acids, and metal ions, playing a crucial role in mediating immune cell homeostasis under to response to different pathological conditions. Increasingly emerging evidence suggests the solute carrier transporters to influence immune cell decision-making, manipulate lymphocyte signaling and its differentiation, function, and fate by mediating different metabolic pathways and different metabolite balance levels [32–34]. Subsequently, we attempted to determine its potential mechanism of action by analyzing the SLC35A2 expression in different immune and molecular subtypes of STAD. Our results robustly indicated the highly different expression of SLC35A2 in different immune and molecular subtypes in STAD, validating SLC35A2 to serve as a reliable diagnostic biomarker and play a role in immune regulation. As a consequence, we employed the TIMER 2.0 and TISIDB to explore the potential capability of SLC35A2 to regulate immune cell recruitment and activation in STAD. Notably, it was clearly elucidated that the SLC35A2 expression was strongly relate to CD4+T cells and macrophages.

On the one hand, the expression level of SLC35A2 exhibited a weak positive correlation with the infiltration level of CD4+T cells, and the correlation suggested the role of SLC35A2 in the regulation of STAD tumor immunology. On the other hand, recent studies have demonstrated that macrophages as a critical immune component of tumor immune microenvironment could function with close association to malignant biological behaviors such as tumor cell proliferation, invasion and immune escape in many reports [35–37]. In the present study, we further revealed the closely relation of macrophage infiltration to the prognosis of STAD patients and a weak correlation between SLC35A2 levels and macrophage-specific markers, including IRF5, CCL2, and CD68. According to these results, we hypothesized a role of SLC35A2 in the process of cell metabolism and macrophage polarization during STAD progression, thereby promoting the malignant bioactivity of STAD cells. Through literature search, we found that a number of bioinformatics studies have reported the prognostic value of SLC family gene expression in different cancers and its potential correlations with immune infiltration [38–40]. Thus, the function of SLC35A2 is closely related to the infiltration of CD4+T cells and macrophages in the tumor microenvironment, which is expected to serve as a potential immune-related marker in STAD tumors.

Metabolic reprogramming of cancer cells, referring to the process by which cancer cells autonomously manipulate their fluxes to meet their bioenergy and biosynthetic requirements through various metabolic pathways, has been a major topic in cancer research in recent years. Purines as the most abundant metabolic substrates in organisms could provide essential energy and cofactors for cell growth and proliferation, in addition to a the composition of DNA and RNA, which means a wide involvement of purines and their derivatives in multiple processes of biological metabolic responses, including immune responses and host-tumor interactions [41]. It is noteworthy that a high concentration of purine metabolites and purinosome in tumor cells may be closely related to the cell cycle [42,43], mediating the cell cycle and enhance the sensitivity to cancer chemotherapy drugs.

As a result, another crucial finding in our analysis was the enrichment of SLC35A2 and many co-expressed genes mainly in the purine metabolism and purinosome, including the asparagine N-linked glycosylation, protein processing in endoplasmic reticulum, metabolism of RNA, SUMOylation of DNA replication proteins, diseases associated with glycosylation precursor biosynthesis, endoplasmic reticulum stress response in coronavirus infection. Moreover, these genes may be involved in VEGFA-VEGFR2 signaling pathway of classical cancer angiogenesis. In recent years, increasing studies have demonstrated the VEGFA-VEGFR2 signaling pathway as one of the significant regulatory mechanisms of tumor angiogenesis, which could regulate the angiogenesis of tumor cells and the energy supply of cancer cells by activated or silenced by certain gene mutations or blocking agents [44,45]. Overall, the results above largely support the role of SLC35A2 gene in cancer cell metabolism, which is expected to be a potential new target for regulating cell metabolism to control cancer cell proliferation. Interestingly, it has been increasingly recognized that in addition to the cancer cells to achieve metabolic pathway changes by driving mutations, other cell types in the tumor microenvironment (endothelial cells, immune cells, fibroblasts) can also contribute to tumorigenesis and progression by alterations in metabolic pathways [46–48]. In the present report, the close relationship between SLC35A2 and immune cells (especially macrophages and CD4+T lymphocytes) was also revealed in the tumor microenvironment. These results to a large extent suggest that SLC35A2 may also regulate the metabolic activities of other cells in the tumor microenvironment, including immune cells and endothelial cells as is closely related to the metabolism of cancer cells, so as to promote the proliferation and angiogenesis of cancer cells.

Despite the systematically analysis of SLC35A2 in this study, there still exist the following shortcomings: Firstly, in vivo and in vitro validation should be carried out to determine the clinical validity of SLC35A2 in patient prognosis and the mechanism of promoting tumor. Secondly, the involvement of multiple databases for analysis could not avoid the bias in sample sources and statistical methods. Summarily, SLC35A2 may be employed as a reliable prognostic marker for gastric cancer patients, with the potential to serve as an important metabolic regulator in cancer cells and immune cells, which is expected to become a new metabolic and immune target for cancer treatment.

## Conclusions

Our study has provided new insights that SLC35A2 could serve as a prognostic biomarker as is correlated with immune cell infiltration in STAD.

## Supporting information

S1 Fig Effects of tumor stage, immune subtypes, molecular subtypes on SLAC35A2 expression. (A) Effects of tumor stage on SLAC35A2 expression. (B, C) Effects of immune subtypes, molecular subtypes on SLAC35A2 expression.
(TIF)

S2 Fig Correlation of SLC35A2 expression with tumor mutation burden (TMB) and microsatellite instability (MSI) in multiple cancer. (A) Correlation between TMB and SLC35A2 expression. (B) Correlation between MSI and SLC35A2 expression.
(TIF)

S1 Dataset (PDF)

S1 File (TXT)

S2 File (XLSX)

S3 File (XLSX)

## Abbreviations

BRCA: breast cancer
DFS: disease-free survival
FP: first-progression
GEPIA: Gene Expression Profiling Interactive Analysis
GO: Gene Ontology
KEGG: Kyoto Encyclopedia of Genes and Genomes
MSI: microsatellite instability
OS: overall survival
PPS: post-progression survival
SLC35A2: solute carrier family 35 member A2
STAD: stomach adenocarcinoma
TMB: tumor mutational burden

## References

[1] Freddie B, Jacques F, Isabelle S ea. Erratum: Global cancer statistics 2018: GLOBOCAN estimates of incidence and mortality worldwide for 36 cancers in 185 countries. CA: a cancer journal for clinicians 2020; 70: 313. 2020/08/09. doi: 10.3322/caac.21609 32767693

[2] Smyth EC, Nilsson M, Grabsch HI, et al. Gastric cancer. Lancet (London, England) 2020; 396: 635–648. 2020/08/31. doi: 10.1016/S0140-6736(20)31288-5 32861308

[3] Bang YJ, Van Cutsem E, Feyereislova A, et al. Trastuzumab in combination with chemotherapy versus chemotherapy alone for treatment of HER2-positive advanced gastric or gastro-oesophageal junction cancer (ToGA): a phase 3, open-label, randomised controlled trial. Lancet (London, England) 2010; 376: 687–697. 2010/08/24. doi: 10.1016/S0140-6736(10)61121-X 20728210

[4] Wilke H, Muro K, Van Cutsem E, et al. Ramucirumab plus paclitaxel versus placebo plus paclitaxel in patients with previously treated advanced gastric or gastro-oesophageal junction adenocarcinoma (RAINBOW): a double-blind, randomised phase 3 trial. The Lancet Oncology 2014; 15: 1224–1235. 2014/09/23. doi: 10.1016/S1470-2045(14)70420-6 25240821

[5] Ng BG, Buckingham KJ, Raymond K, et al. Mosaicism of the UDP-galactose transporter SLC35A2 causes a congenital disorder of glycosylation. American journal of human genetics 2013; 92: 632–636. 2013/04/09. doi: 10.1016/j.ajhg.2013.03.012 23561849 PMC3617373

[6] Song Z. Roles of the nucleotide sugar transporters (SLC35 family) in health and disease. Molecular aspects of medicine 2013; 34: 590–600. 2013/03/20. doi: 10.1016/j.mam.2012.12.004 23506892

[7] Kodera H, Nakamura K, Osaka H, et al. De novo mutations in SLC35A2 encoding a UDP-galactose transporter cause early-onset epileptic encephalopathy. Human mutation 2013; 34: 1708–1714. 2013/10/12. doi: 10.1002/humu.22446 24115232

[8] Dörre K, Olczak M, Wada Y, et al. A new case of UDP-galactose transporter deficiency (SLC35A2-CDG): molecular basis, clinical phenotype, and therapeutic approach. Journal of inherited metabolic disease 2015; 38: 931–940. 2015/03/18. doi: 10.1007/s10545-015-9828-6 25778940

[9] Yates TM, Suri M, Desurkar A, et al. SLC35A2-related congenital disorder of glycosylation: Defining the phenotype. European journal of paediatric neurology: EJPN: official journal of the European Paediatric Neurology Society 2018; 22: 1095–1102. 2018/09/09. doi: 10.1016/j.ejpn.2018.08.002 30194038

[10] Girardi E, César-Razquin A, Lindinger S, et al. A widespread role for SLC transmembrane transporters in resistance to cytotoxic drugs. Nature chemical biology 2020; 16: 469–478. 2020/03/11. doi: 10.1038/s41589-020-0483-3 32152546 PMC7610918

[11] Ta HDK, Minh Xuan DT, Tang WC, et al. Novel Insights into the Prognosis and Immunological Value of the SLC35A (Solute Carrier 35A) Family Genes in Human Breast Cancer. Biomedicines 2021; 9 2021/12/25. doi: 10.3390/biomedicines9121804 34944621 PMC8698499

[12] Li T, Fan J, Wang B, et al. TIMER: A Web Server for Comprehensive Analysis of Tumor-Infiltrating Immune Cells. Cancer research 2017; 77: e108–e110. 2017/11/03. doi: 10.1158/0008-5472.CAN-17-0307 29092952 PMC6042652

[13] Li B, Severson E, Pignon JC, et al. Comprehensive analyses of tumor immunity: implications for cancer immunotherapy. Genome biology 2016; 17: 174. 2016/08/24. doi: 10.1186/s13059-016-1028-7 27549193 PMC4993001

[14] Ru B, Wong CN, Tong Y, et al. TISIDB: an integrated repository portal for tumor-immune system interactions. Bioinformatics (Oxford, England) 2019; 35: 4200–4202. 2019/03/25. doi: 10.1093/bioinformatics/btz210 30903160

[15] Tang Z, Li C, Kang B, et al. GEPIA: a web server for cancer and normal gene expression profiling and interactive analyses. Nucleic acids research 2017; 45: W98–w102. 2017/04/14. doi: 10.1093/nar/gkx247 28407145 PMC5570223

[16] Chandrashekar DS, Bashel B, Balasubramanya SAH, et al. UALCAN: A Portal for Facilitating Tumor Subgroup Gene Expression and Survival Analyses. Neoplasia (New York, NY) 2017; 19: 649–658. 2017/07/22. doi: 10.1016/j.neo.2017.05.002 28732212 PMC5516091

[17] Lánczky A and Győrffy B. Web-Based Survival Analysis Tool Tailored for Medical Research (KMplot): Development and Implementation. Journal of medical Internet research 2021; 23: e27633. 2021/07/27. doi: 10.2196/27633 34309564 PMC8367126

[18] Koch A, Jeschke J, Van Criekinge W, et al. MEXPRESS update 2019. Nucleic acids research 2019; 47: W561–w565. 2019/05/23. doi: 10.1093/nar/gkz445 31114869 PMC6602516

[19] Gao J, Aksoy BA, Dogrusoz U, et al. Integrative analysis of complex cancer genomics and clinical profiles using the cBioPortal. Science signaling 2013; 6: pl1. 2013/04/04. doi: 10.1126/scisignal.2004088 23550210 PMC4160307

[20] Vasaikar SV, Straub P, Wang J, et al. LinkedOmics: analyzing multi-omics data within and across 32 cancer types. Nucleic acids research 2018; 46: D956–d963. 2017/11/15. doi: 10.1093/nar/gkx1090 29136207 PMC5753188

[21] Zhou Y, Zhou B, Pache L, et al. Metascape provides a biologist-oriented resource for the analysis of systems-level datasets. Nature communications 2019; 10: 1523. 2019/04/05. doi: 10.1038/s41467-019-09234-6 30944313 PMC6447622

[22] Matsuoka T and Yashiro M. Biomarkers of gastric cancer: Current topics and future perspective. World journal of gastroenterology 2018; 24: 2818–2832. 2018/07/19. doi: 10.3748/wjg.v24.i26.2818 30018477 PMC6048430

[23] de Castro Sant’ Anna C, Junior AGF, Soares P, et al. Molecular biology as a tool for the treatment of cancer. Clinical and experimental medicine 2018; 18: 457–464. 2018/07/15. doi: 10.1007/s10238-018-0518-1 30006681

[24] Liu CL, Cheng SP, Huang WC, et al. Aberrant Expression of Solute Carrier Family 35 Member A2 Correlates With Tumor Progression in Breast Cancer. In vivo (Athens, Greece) 2023; 37: 262–269. 2023/01/03. doi: 10.21873/invivo.13076 36593004 PMC9843756

[25] Yin Y, Jiang Z, Fu J, et al. Choline-induced SLC5A7 impairs colorectal cancer growth by stabilizing p53 protein. Cancer letters 2022; 525: 55–66. 2021/09/26. doi: 10.1016/j.canlet.2021.09.027 34562520

[26] Goodman AM, Kato S, Bazhenova L, et al. Tumor Mutational Burden as an Independent Predictor of Response to Immunotherapy in Diverse Cancers. Molecular cancer therapeutics 2017; 16: 2598–2608. 2017/08/25. doi: 10.1158/1535-7163.MCT-17-0386 28835386 PMC5670009

[27] Samstein RM, Lee CH, Shoushtari AN, et al. Tumor mutational load predicts survival after immunotherapy across multiple cancer types. Nature genetics 2019; 51: 202–206. 2019/01/16. doi: 10.1038/s41588-018-0312-8 30643254 PMC6365097

[28] Mouradov D, Domingo E, Gibbs P, et al. Survival in stage II/III colorectal cancer is independently predicted by chromosomal and microsatellite instability, but not by specific driver mutations. The American journal of gastroenterology 2013; 108: 1785–1793. 2013/09/18. doi: 10.1038/ajg.2013.292 24042191

[29] Mandal R, Samstein RM, Lee KW, et al. Genetic diversity of tumors with mismatch repair deficiency influences anti-PD-1 immunotherapy response. Science (New York, NY) 2019; 364: 485–491. 2019/05/03. doi: 10.1126/science.aau0447 31048490 PMC6685207

[30] Chen Y, Li S, Brown C, et al. Effect of genetic variation in the organic cation transporter 2 on the renal elimination of metformin. Pharmacogenetics and genomics 2009; 19: 497–504. 2009/06/02. doi: 10.1097/FPC.0b013e32832cc7e9 19483665 PMC3104496

[31] Chen L, Shu Y, Liang X, et al. OCT1 is a high-capacity thiamine transporter that regulates hepatic steatosis and is a target of metformin. Proceedings of the National Academy of Sciences of the United States of America 2014; 111: 9983–9988. 2014/06/26. doi: 10.1073/pnas.1314939111 24961373 PMC4103324

[32] Song W, Li D, Tao L, et al. Solute carrier transporters: the metabolic gatekeepers of immune cells. Acta pharmaceutica Sinica B 2020; 10: 61–78. 2020/01/30. doi: 10.1016/j.apsb.2019.12.006 31993307 PMC6977534

[33] Frauwirth KA and Thompson CB. Regulation of T lymphocyte metabolism. Journal of immunology (Baltimore, Md: 1950) 2004; 172: 4661–4665. 2004/04/07. doi: 10.4049/jimmunol.172.8.4661 15067038

[34] Kavanagh Williamson M, Coombes N, Juszczak F, et al. Upregulation of Glucose Uptake and Hexokinase Activity of Primary Human CD4+ T Cells in Response to Infection with HIV-1. Viruses 2018; 10 2018/03/10. doi: 10.3390/v10030114 29518929 PMC5869507

[35] Lin Y, Xu J and Lan H. Tumor-associated macrophages in tumor metastasis: biological roles and clinical therapeutic applications. Journal of hematology & oncology 2019; 12: 76. 2019/07/14. doi: 10.1186/s13045-019-0760-3 31300030 PMC6626377

[36] Mantovani A, Marchesi F, Malesci A, et al. Tumour-associated macrophages as treatment targets in oncology. Nature reviews Clinical oncology 2017; 14: 399–416. 2017/01/25. doi: 10.1038/nrclinonc.2016.217 28117416 PMC5480600

[37] Wang J, Li D, Cang H, et al. Crosstalk between cancer and immune cells: Role of tumor-associated macrophages in the tumor microenvironment. Cancer medicine 2019; 8: 4709–4721. 2019/06/22. doi: 10.1002/cam4.2327 31222971 PMC6712467

[38] Li J, Xie J, Wu D, et al. A pan-cancer analysis revealed the role of the SLC16 family in cancer. Channels (Austin, Tex) 2021; 15: 528–540. 2021/08/24. doi: 10.1080/19336950.2021.1965422 34424811 PMC8386723

[39] Xue L, Liu J, Xie J, et al. Prognostic Value of SLC16A3(MCT4) in Lung Adenocarcinoma and Its Clinical Significance. International journal of general medicine 2021; 14: 8413–8425. 2021/11/26. doi: 10.2147/IJGM.S337615 34819749 PMC8607606

[40] Xie J, Ruan S, Zhu Z, et al. Database mining analysis revealed the role of the putative H(+)/sugar transporter solute carrier family 45 in skin cutaneous melanoma. Channels (Austin, Tex) 2021; 15: 496–506. 2021/08/03. doi: 10.1080/19336950.2021.1956226 34334114 PMC8331014

[41] Di Virgilio F and Adinolfi E. Extracellular purines, purinergic receptors and tumor growth. Oncogene 2017; 36: 293–303. 2016/06/21. doi: 10.1038/onc.2016.206 27321181 PMC5269532

[42] An S, Kumar R, Sheets ED, et al. Reversible compartmentalization of de novo purine biosynthetic complexes in living cells. Science (New York, NY) 2008; 320: 103–106. 2008/04/05. doi: 10.1126/science.1152241 18388293

[43] Chan CY, Zhao H, Pugh RJ, et al. Purinosome formation as a function of the cell cycle. Proceedings of the National Academy of Sciences of the United States of America 2015; 112: 1368–1373. 2015/01/22. doi: 10.1073/pnas.1423009112 25605889 PMC4321313

[44] Xu X, Wu L, Zhou X, et al. Cryptotanshinone inhibits VEGF-induced angiogenesis by targeting the VEGFR2 signaling pathway. Microvascular research 2017; 111: 25–31. 2017/01/04. doi: 10.1016/j.mvr.2016.12.011 28040437

[45] Sadremomtaz A, Mansouri K, Alemzadeh G, et al. Dual blockade of VEGFR1 and VEGFR2 by a novel peptide abrogates VEGF-driven angiogenesis, tumor growth, and metastasis through PI3K/AKT and MAPK/ERK1/2 pathway. Biochimica et biophysica acta General subjects 2018; 1862: 2688–2700. 2018/09/27. doi: 10.1016/j.bbagen.2018.08.013 30251659

[46] Martínez-Reyes I and Chandel NS. Cancer metabolism: looking forward. Nature reviews Cancer 2021; 21: 669–680. 2021/07/18. doi: 10.1038/s41568-021-00378-6 34272515

[47] Ryan DG and O’Neill LAJ. Krebs Cycle Reborn in Macrophage Immunometabolism. Annual review of immunology 2020; 38: 289–313. 2020/01/28. doi: 10.1146/annurev-immunol-081619-104850 31986069

[48] Makowski L, Chaib M and Rathmell JC. Immunometabolism: From basic mechanisms to translation. Immunological reviews 2020; 295: 5–14. 2020/04/23. doi: 10.1111/imr.12858 32320073 PMC8056251

