# Supplementary figures and images for: Solute carrier family 35 member A2 (SLC35A2) is a prognostic biomarker and correlated with immune infiltration in stomach adenocarcinoma
Source: PLoS One. 2023 Jul 19;18(7):e0287303. doi: 10.1371/journal.pone.0287303 (PMC10355401; doi:10.1371/journal.pone.0287303)

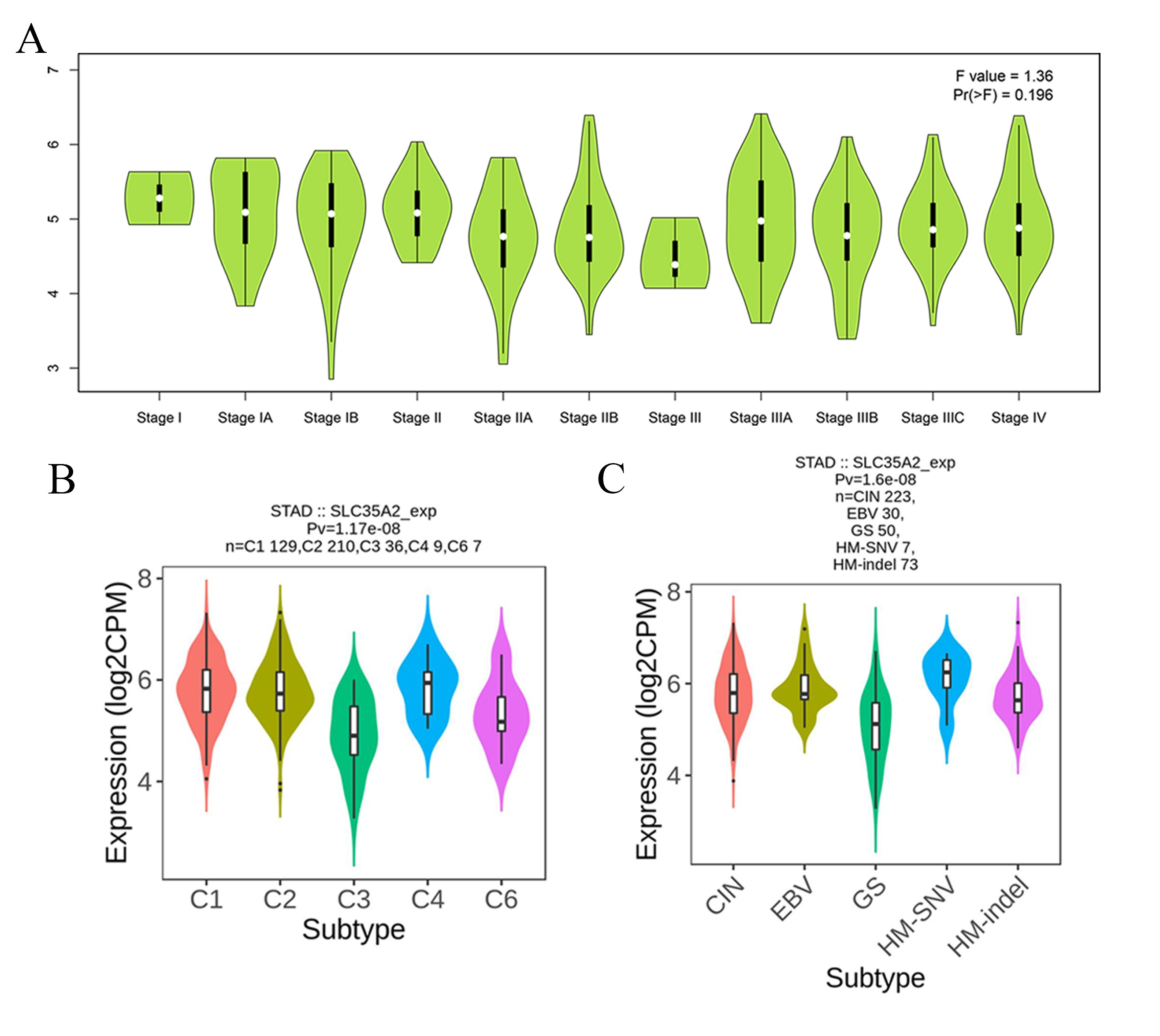

Supplement: S1 Fig — (A) Effects of tumor stage on SLAC35A2 expression. (B, C) Effects of immune subtypes, molecular subtypes on SLAC35A2 expression. (TIF) [file pone.0287303.s001.tif]

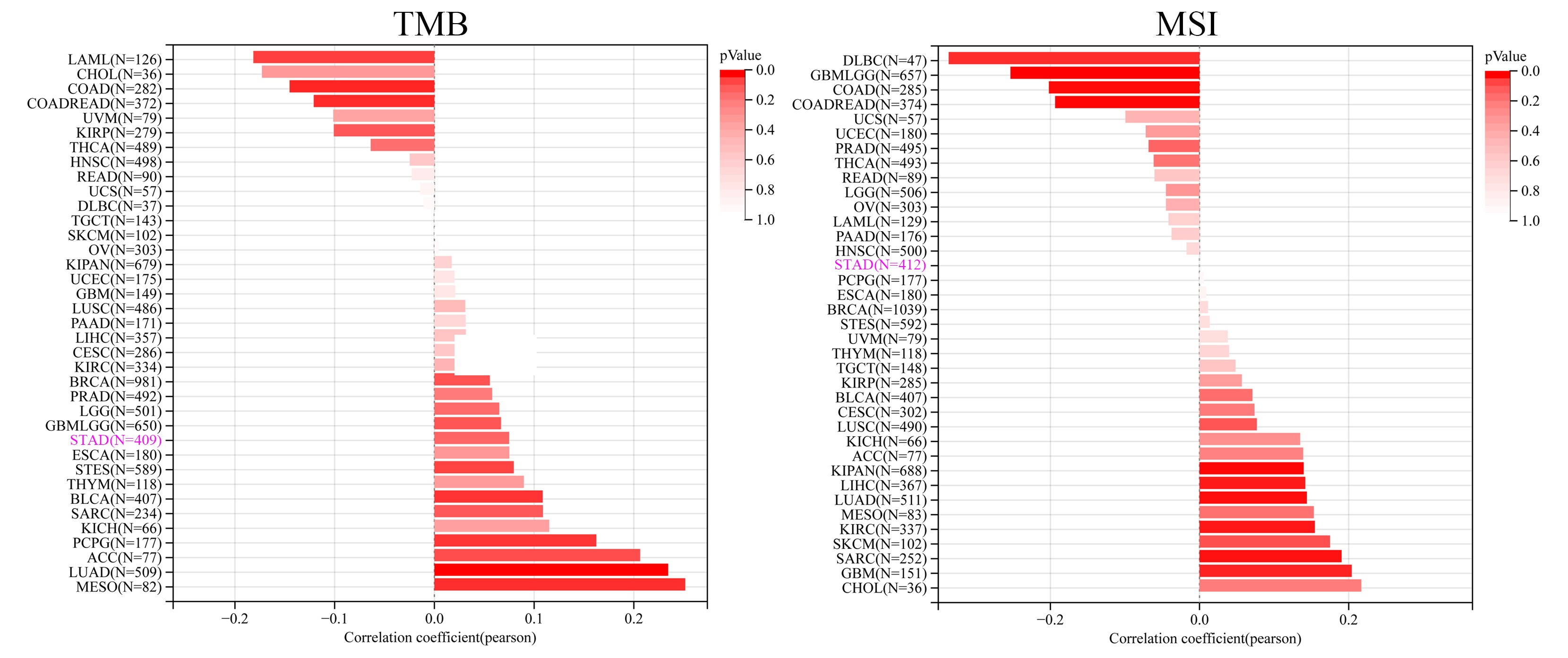

Supplement: S2 Fig — (A) Correlation between TMB and SLC35A2 expression. (B) Correlation between MSI and SLC35A2 expression. (TIF) [file pone.0287303.s002.tif]
