# Supplementary material for: Solute carrier family 35 member A2 (SLC35A2) is a prognostic biomarker and correlated with immune infiltration in stomach adenocarcinoma
Source: PLoS One. 2023 Jul 19;18(7):e0287303. doi: 10.1371/journal.pone.0287303 (PMC10355401; doi:10.1371/journal.pone.0287303)
Supplement: S1 Dataset — (PDF) [file pone.0287303.s003.pdf]

Minimal data set of the paper "Solute carrier family 35 member A2 (SLC35A2) is a prognostic biomarker and correlated with immune infiltration in stomach adenocarcinoma"

Note: The data in this paper was last analyzed on February 5, 2023. Since the online database will be updated from time to time, the reproduced data may be different.

1. TIMER, https://cistrome.shinyapps.io/timer/

(1) Follow a step-by-step example analysis.

(2) Enter "SLC35A2" in "Diff Exp module" (Fig 1A).

(3) In "Gene module", enter the target gene "SLC35A2", select a cancer type "STAD" and submit (Fig 6A).

(4) In "Survival module", select a cancer type "STAD", enter the target gene "SLC35A2" (Fig 6B).

(5) In "SCNA module", select a cancer type "STAD", enter the target gene "SLC35A2", and submit for analysis (Table 2).

(6) In "Correlation module", select a cancer type "STAD", enter the target gene "SLC35A2" and gene markers of various immune cells, select "No" or "Tumor Purity", respectively, and submit for analysis (Fig 6D).

2. **TISIDB, http://cis.hku.hk/TISIDB/**

(1) Follow a step-by-step example analysis.

(2) In "Quick search module", enter the target gene "SLC35A2" and submit.

(3) Browse "Subtype" module, obtain the S1_FigB-C.

(4) Browse "Lymphocyte" module, select a cancer type "STAD" and select an item (Fig 6C, Table 3).

3. **GEPIA, http://gepia.cancer-pku.cn/**

(1) Follow a step-by-step example analysis.

(2) In "Boxplots" module, enter the target gene "SLC35A2" and select a cancer type "STAD" (Fig 1B). Enter the target gene "PMM2", "FTSJ1" and select a cancer type "STAD" (Fig 5A, D).

(3) In "Survival Plots" module, enter the target gene "SLC35A2" and select a cancer type "STAD", select "OS" or "RFS", respectively, and submit for analysis (Fig 1F, G).

(4) In "Correlation" module, enter the target gene "SLC35A2" and "PMM2", "FTSJ1", select a cancer type "STAD" and a normal type "STAD" (Fig 5B, E).

4. **UALCAN, http://ualcan.path.uab.edu/index.html**

(1) Follow a step-by-step example analysis.

(2) In "TCGA" module, enter gene symbol "SLC35A2" and select

a cancer type “STAD”, browse the expression, methylation of target gene “SLC35A2” (Fig 1C, D).

(3) In “CPTAC” module, enter gene symbol “SLC35A2” and select a cancer type “STAD”, browse the protein expression of target gene “SLC35A2” (Fig 1E). Based on “sample types”, explore the expression level of SLC35A2 (Fig 2).

5. SangerBox, http://sangerbox.com

(1) Follow a step-by-step example analysis.

(2) In “Pan-cancer Analysis” module, TMB and MSI were analyzed in Genome Heterogeneity and gene expression analysis (S2 Fig).

6. Kaplan–Meier plotter, https://kmplot.com/analysis/

(1) Follow a step-by-step example analysis.

(2) Select the “Gastric cancer” module, enter gene symbol “SLC35A2” and explore the relationships between SLC35A2 expression and prognosis, including OS, FP, PPS (Fig 1H-J). Based on different clinical characteristics, analyze the correlations between SLC35A2 expression and OS, FP, PPS (Table 1).

7. MEXPRESS, https://mexpress.be/index.html

(1) Follow a step-by-step example analysis.

(2) Enter a gene or miRNA name “SCLS35A2”.

(3) Select a cancer type “STAD” and click the “PLOT” (Fig 3A).

8. cBioPortal, http://www.cbioportal.org/

(1) In “Query” module, select the stomach tissue and data sets (TCGA, Nuture 2014; TCGA, PanCancer Atlas; TCGA, Firehose Legacy), click the “Query By Gene” and enter the target gene “SCLS35A2”. Browse “Cancer Types Summary”, “Mutations”, “Comparison/Survival” modules (Fig 3B-G).

(2) In “Query” module, select the stomach tissue and data sets (TCGA, Nuture 2014; TCGA, PanCancer Atlas; TCGA, Firehose Legacy), click the “Query By Gene” and enter the 15 co-expressed genes (TIMM17B, APEX2, FTSJ1, RPN1, PRICKLE3, PMM2, SURF4, RPN2, LRRC59, SEC61A1, TMED9, SRPRB, PYCR1, SLC39A7, CDK16). Browse “OncoPrint” module (Fig 3F).

(3) Browse the “Comparison/Survival” module and select the “Genomic Alterations” and enter the 15 co-expressed genes (TIMM17B, APEX2, FTSJ1, RPN1, PRICKLE3, PMM2, SURF4, RPN2, LRRC59, SEC61A1, TMED9, SRPRB, PYCR1, SLC39A7, CDK16) to analyze the differences in expression between cancer and normal tissues (Fig 4E).

9. LinkedOmics, http://www.linkedomics.org/admin.php

(1) Follow a step-by-step example analysis.

(2) Step 1, select “TCGA_STAD”; Step 2, select data type “RNAseq”; Step 3, enter the target gene “SCLS35A2”; Step 4, select data type “RNAseq”; Step 4, select the statistical method “Pearson Correlation test” and submit (Fig 4A-C).

10. Metascape, https://metascape.org

(1) Step 1, SLC35A2-related genes from GEPIA and LinkedOmics databases were enrolled into Metascape analysis platform; Step 2, select “Any Species”, “H. sapiens”; Step 3, click the “Express Analysis” (Fig 5G-H).
